# The 10 year safety and efficacy of tenofovir disoproxil fumarate (TDF)-containing once-daily highly active antiretroviral therapy (HAART)

**DOI:** 10.1186/1758-2652-13-S4-P86

**Published:** 2010-11-08

**Authors:** I Cassetti, A Etzel, JV Madruga, JM Suleiman, Y Zhou, M Rhee, DR Warren

**Affiliations:** 1 Fundacion Centro Estudios Infectologicos, Buenos Aires, Argentina; 2 Hospital Guilherme Álvaro, Santos, Brazil; 3 Centro de Referencia e Treinamento DST/AIDS, Sao Paulo, Brazil; 4 Brasilmed Assistência Médica e Pesquisas, Sao Paulo, Brazil; 5 Gilead Sciences, Inc, Foster City, USA

## Background

Study 903 was a Phase III randomized double-blind (DB) 3 year study comparing TDF to stavudine (d4T) each in combination with lamivudine (3TC) and efavirenz (EFV) in HIV-1 infected antiretroviral naïve patients. TDF was associated with durable efficacy and safety (better lipid profile, and less lipodystrophy and peripheral neuropathy). A subset of these patients now provides 10 years of longitudinal efficacy and safety data of TDF-containing once-daily HAART.

## Methods

Subjects in Argentina, Brazil, and the Dominican Republic who completed the 3 year DB period of study were eligible to roll-over into an open-label (OL) study (Study 903E) of the once-daily HAART regimen, TDF+3TC+EFV. At DB baseline 86 subjects were randomized to TDF (62% male, 70% white, mean age 33 yrs, mean HIV RNA=4.9 log_10_ c/mL, and mean CD4 count=299 cells/mm^3^). At OL baseline, 85 subjects (60% male, 64% white, mean age 37 yrs, median CD4=621 cells/mm^3^) switched from d4T to TDF. The results reflect only the period of TDF exposure.

## Results

See Table 1

**Table 1:** 

	TDF/TDF^α^ (n=86)	D4T/TDF^α^ (n=85)
Weeks on HAART/TDF	480/480	480/336

HIV RNA < 50 (copies/mL) at Week 480 (ITT, M=F)	63%	64%

HIV RNA < 50 (copies/mL) at Week 480 (ITT, M=E)	92%	96%

Change in Mean (SD) CD4, cells/mm^3^	545 (287)	180 (290)

Drug-related Adverse Events (Grades 1-4)	66%	46%

Change in Mean (SD) Creatinine Clearance, mL/min^β^	+2.5 (23.4)	-10.7 (22.6)

Median Limb Fat at Year 10, kg	10.4	7.5

Percent Change in Mean (SD) Spine BMD^χ^	-2.44 (5.08)^δ^	0.04 (4.72)

Percent Change in Mean (SD) Hip BMD	-2.94 (4.95)^δ^	-1.86 (4.67)^δ^

Discontinuations during open-label extension	25 (29.1%)	19 (22.4%)

Adverse event	2 (2.3%)	2 (2.4%)

Suboptimal virologic response	5 (5.8%)	1 (1.2%)

LTFU^ε^, Nonadherent, Pregnancy, Consent Withdrawn, Death	13 (15.1%)	9 (10.6%)

Other	5 (5.8%)	7 (8.2%)

^α^TDF/TDF results measured from DB BL; d4T/TDF from OL baseline; ^β^Estimated by Cockcroft-Gault equation; ^χ^Bone mineral density; ^δ^p<0.01 by Wilcoxon Signed Rank Test; ^ε^Lost to follow-up

## Conclusions

Antiretroviral-naïve subjects who received TDF-containing once-daily HAART for up to 10 years demonstrated sustained virologic and immunologic benefit, improved limb fat, stable renal function, and their BMD remained stable after a clinically insignificant decrease that occurred during the first year of TDF therapy.

